# Boston biorepository, recruitment and integrative network (BBRAIN): A resource for the Gulf War Illness scientific community

**DOI:** 10.1016/j.lfs.2021.119903

**Published:** 2021-08-26

**Authors:** D. Keating, C.G. Zundel, M. Abreu, M. Krengel, K. Aenlle, M.D. Nichols, R. Toomey, L.L. Chao, J. Golier, L. Abdullah, E. Quinn, T. Heeren, J.R. Groh, B.B. Koo, R. Killiany, M.L. Loggia, J. Younger, J. Baraniuk, P. Janulewicz, J. Ajama, M. Quay, P.W. Baas, L. Qiang, L. Conboy, E. Kokkotou, J.P. O'Callaghan, L. Steele, N. Klimas, K. Sullivan

**Affiliations:** a Boston University School of Public Health, Department of Environmental Health, 715 Albany St. T4W, Boston, MA 02118, USA; b Boston University School of Medicine, Behavioral Neuroscience Program, 72 East Concord St., Boston, MA 02118, USA; c Dr. Kiran C. Patel College of Osteopathic Medicine, Institute for Neuroimmune Medicine, Nova Southeastern University, Fort Lauderdale, FL 33314, USA; d Geriatric Research Education and Clinical Center, Miami VA Medical Center, Miami, FL 33125, USA; e Boston University School of Medicine, Department of Neurology, 72 East Concord St., Boston, MA 02118, USA; f Department of Psychological and Brain Sciences, College of Arts and Sciences, Boston University, 900 Commonwealth Ave., Boston, MA, USA; g San Francisco Veterans Affairs Health Care System, University of California, San Francisco, CA 94143, USA; h James J. Peters VA Medical Center, OOMH-526, 130 West Kingsbridge Road, Bronx, NY 10468, USA; i Psychiatry Department, Icahn School of Medicine at Mount Sinai, 1428 Madison Ave, New York, NY 10029, USA; j Roskamp Institute, 2040 Whitfield Ave, Sarasota, FL 34243, USA; k Open University, Milton Keynes, United Kingdom; l James A. Haley Veterans' Hospital, Tampa, FL, USA; m Boston University School of Public Health, Department of Biostatistics, 715 Albany St., Boston, MA 02118, USA; n Boston University School of Medicine, Department of Anatomy and Neurobiology, 72 East Concord St., Boston, MA 02118, USA; o Department of Radiology, Athinoula A. Martinos Center for Biomedical Imaging, Massachusetts General Hospital, Harvard Medical School, Charlestown, MA, USA; p Neuroinflammation, Pain & Fatigue Lab, University of Alabama at Birmingham, Birmingham, AL, USA; q Department of Medicine, Georgetown University, Washington, DC, USA; r Drexel University College of Medicine, Department of Neurobiology and Anatomy, 2900 Queen Lane, Philadelphia, PA 19129, USA; s Department of Medicine, Beth Israel Deaconess Medical Center, Harvard Medical School, Boston, MA 02115, USA; t Health Effects Laboratory Division, Centers for Disease Control and Prevention, National Institute for Occupational Safety and Health, Morgantown, WV, USA; u Baylor College of Medicine Neuropsychiatry Division, Department of Psychiatry and Behavioral Sciences, Houston, TX 77030, USA

**Keywords:** BBRAIN, GWI, GWIC, Gulf war, Gulf war illness

## Abstract

**Aims:**

Gulf War Illness (GWI), a chronic debilitating disorder characterized by fatigue, joint pain, cognitive, gastrointestinal, respiratory, and skin problems, is currently diagnosed by self-reported symptoms. The Boston Biorepository, Recruitment, and Integrative Network (BBRAIN) is the collaborative effort of expert Gulf War Illness (GWI) researchers who are creating objective diagnostic and pathobiological markers and recommend common data elements for GWI research.

**Main methods:**

BBRAIN is recruiting 300 GWI cases and 200 GW veteran controls for the prospective study. Key data and biological samples from prior GWI studies are being merged and combined into retrospective datasets. They will be made available for data mining by the BBRAIN network and the GWI research community. Prospective questionnaire data include general health and chronic symptoms, demographics, measures of pain, fatigue, medical conditions, deployment and exposure histories. Available repository biospecimens include blood, plasma, serum, saliva, stool, urine, human induced pluripotent stem cells and cerebrospinal fluid.

**Key findings:**

To date, multiple datasets have been merged and combined from 15 participating study sites. These data and samples have been collated and an online request form for repository requests as well as recommended common data elements have been created. Data and biospecimen sample requests are reviewed by the BBRAIN steering committee members for approval as they are received.

**Significance:**

The BBRAIN repository network serves as a much needed resource for GWI researchers to utilize for identification and validation of objective diagnostic and pathobiological markers of the illness.

## 1. Introduction

Gulf War Illness (GWI) is a debilitating, chronic, multi-symptom disorder affecting nearly one-third of veterans in the 1991 Gulf War (GW) [1,2]. The illness is characterized by debilitating fatigue, chronic pain, cognitive dysfunction, headaches, respiratory problems, and gastrointestinal disturbances [3-5]. Veterans suffering from GWI can experience significant impairment in their daily activities and quality of life. Despite promising recent research in correlating biomarkers to GWI symptoms, GWI primarily remains diagnosed by self-report. The study of potential diagnostic biomarkers to date has not been supported by larger sample sizes and has not been validated in other cohorts [6-11]. Basing diagnosis on self-reported symptoms makes treatment development and access to care for GW veterans persistently difficult. There is a critical need for an objective diagnostic test for GWI to alleviate GW veterans’ difficulties with obtaining service-related benefits and validation of their symptoms and for use as primary outcome measures for treatment trials.

“A biorepository is an entity that receives, processes, stores, and/or disseminates biospecimens, their derivatives, and relevant data, as needed [12]. It encompasses the physical location and the full range of activities associated with its operation [12].” A recent review by Garcia et al., showed that rare disease biobanks have the ability to identify and validate genetic and omics biomarkers as well as inform treatment development strategies for these rare disorders [13]. However, it was also noted that many of these repositories lacked the corresponding clinical outcomes data needed to make the biomarker samples most useful for correlation with the disease symptoms.

Therefore, the need is clear for a biorepository network of freely sharing biospecimens with corresponding comprehensive clinical outcomes data in the field of GWI research. There is also a need for retrospective data mining from prior studies that are hard to replicate (i.e., cerebrospinal fluid, PET, and MRI brain imaging outcomes). These clinical outcomes that are common (common data elements) among the different prior studies provide power to document differences that might not emerge in the smaller sample cohorts. These common data elements are also needed to ensure comparability of study results, particularly for treatment trial efficacy testing [14,15].

The Boston Biorepository, Recruitment and Integrative Network (BBRAIN) for GWI was designed to serve as a resource for the GWI research community to hasten biomarker discovery and validate prior results in a well-characterized cohort of GW veterans. The BBRAIN study was built upon and incorporates the already existing Boston GWI consortium [16]. The GWI consortium brought together leading experts from different fields into the GWI research community. Since its conception, the GWI consortium has established an extensive multi-site data set with cognitive measures, brain imaging, health symptom data, and biorepository blood and saliva specimens for several hundred GW veterans. GWIC has greatly expanded the field's ability to explore and identify specific ‘objective’ biomarkers and ‘personalized’ treatment strategies for veterans with GWI by utilizing a small biorepository shared with the GWI research community, resulting in 20 additional federally funded studies. This lead to 34 biomarker publications of lipidomic, proteomic, epigenetic, genetic susceptibility, mitochondrial, CNS autoantibodys, and tau markers in clinical and preclinical translational studies [7,11,17-43]. It also resulted in funding to establish two additional consortia, including BBRAIN and the Gulf War Illness Clinical Trials Consortium [14]. Since its inception in 2018, BBRAIN has built upon this existing infrastructure at Boston University and 14 other participating sample and data resource sites to establish a much-needed resource for the GWI research community that is available for data and sample sharing.

## 2. Methods: BBRAIN structure

### 2.1. Leadership

The lead site of BBRAIN is at Boston University School of Public Health and makes up the network coordinating center. The network coordinating center staff members have diverse expertise in neuropsychology, brain imaging, exposure assessment, data management, statistical programming and study operations. These skills are integral for maintaining a multi-site biorepository and promoting collaboration within the GWI research field.

### 2.2. Participating sites

**Fig. 1.**
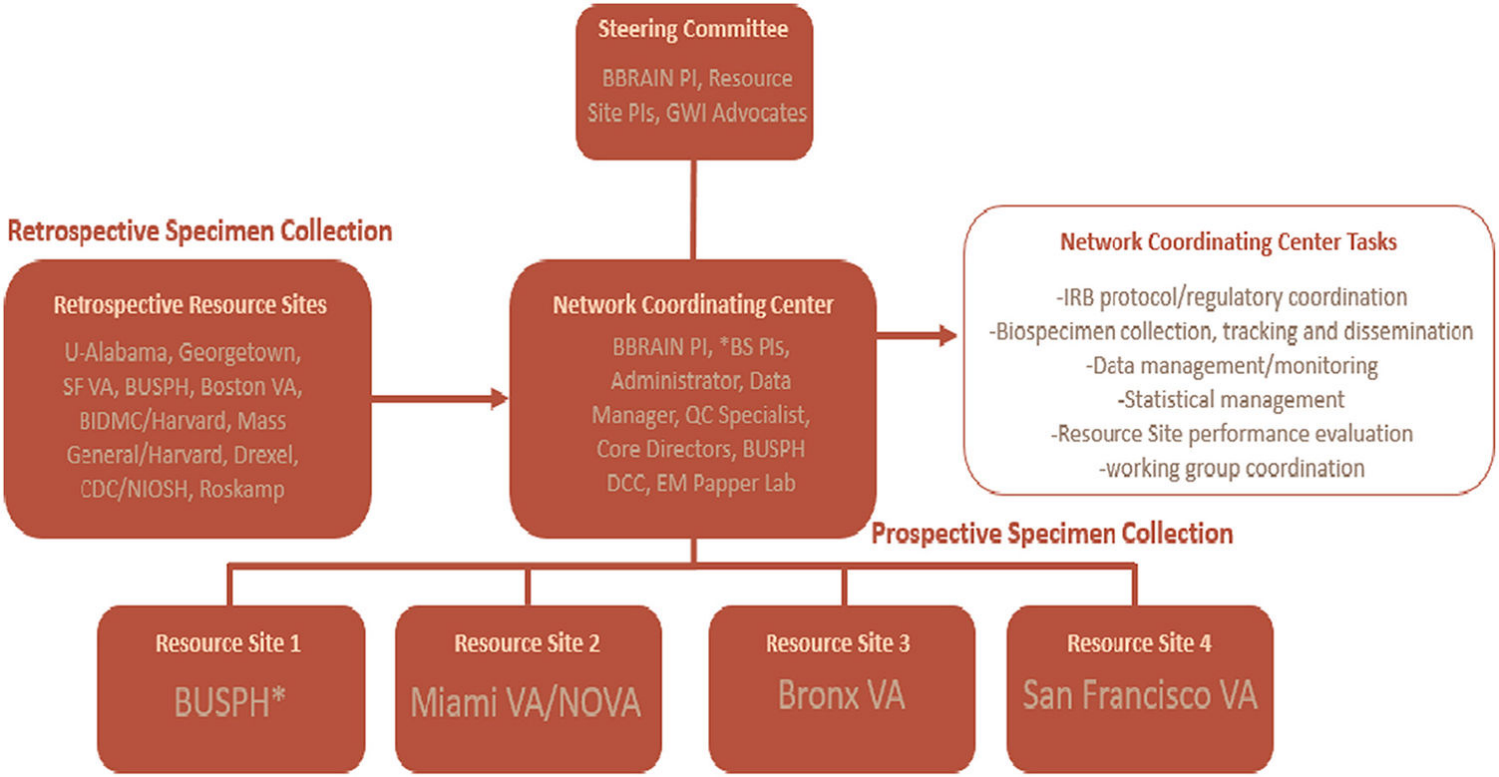
BBRAIN organizational structure. *Resource Site and Biorepository storage site

The BBRAIN collaboration brings together leading investigators from 15 institutions to support participant recruitment, administrative activities, data management and biostatistics, and biorepository and biomarker evaluation. The BBRAIN collaboration is composed of the network coordinating center, steering committee, retrospective resource sites and four prospective resource sites (Fig. 1). The four resource sites where prospective subject recruitment is taking place include Boston University School of Public Health, Miami VA Medical Center and Nova Southeastern University, Bronx VA Medical Center and the San Francisco VA Medical Center.

#### 2.2.1. Steering committee

Oversight of the BBRAIN is coordinated by a Steering Committee made up of the BBRAIN PI, the resource site PIs, the network coordinating directors, and the consumer advocate. The Steering Committee monitors research site performance and determines individual study performance. The Steering Committee is also responsible for establishing standard operating procedures, utilizing ISBER Best Practices for Biorepositories, and utilizing BUSPH criteria templates for Data Use Agreements across sites and institutions sharing samples and data [12]. Researchers interested in obtaining BBRAIN samples can apply to the Steering Committee. The group will decide on the appropriateness (i.e., for GWI research and not redundant with ongoing research) and priority of sharing samples on a case-by-case basis.

#### 2.2.2. Network coordinating center

The network coordinating center for the biorepository is responsible for overseeing IRB protocol and regulatory submissions and approvals, establishing standard protocols across all sites, and conducting data management and monitoring while ensuring study participant confidentiality. The center is led by the BBRAIN study PI at BUSPH and supported by faculty and administrative staff at BUSPH. The network coordinating center provides support and coordination for prospective data collection of demographic surveys, cognitive test data, serum, plasma, saliva, stool, and urine samples from 500 GW veteran study participants. Additionally, the network coordinating center serves as a gatekeeper for requests for repository site sharing and coordinates approvals with the steering committee members in consultation with the biorepository contributors. A virtual biorepository is established using laboratory software LDMS (Frontier and/or FreezerPro for resource sites). Resource sites send newly obtained biospecimens and data to laboratory storage facilities at Nova Southeastern University and Boston University Medical Campus (BUMC) as the prospective repository is being created.

##### 2.2.2.1. Subject Confidentiality.

As in all human subject research, protecting subject confidentiality is imperative. For retrospective data, all samples and data are de-identified. For prospective data collection, participants’ contact information is kept in a study-specific electronic capture web-based platform on a secure server, including multiple password protection layers that are only accessible by approved study team members. All source documents are kept in a locked cabinet, and all data is behind password-protected and encrypted devices. Samples that are shipped are labeled with a unique identifier code for tracking purposes within the biorepository. Participants are made aware of the confidentiality measure taken at the time of the phone screener and again during the consent process and are consented to share their coded study samples and data for the repository and other future GWI related studies. All data and samples that are shared from the repository are coded with no individual identifiers.

#### 2.2.3. Retrospective resource sites

**Fig. 2.**
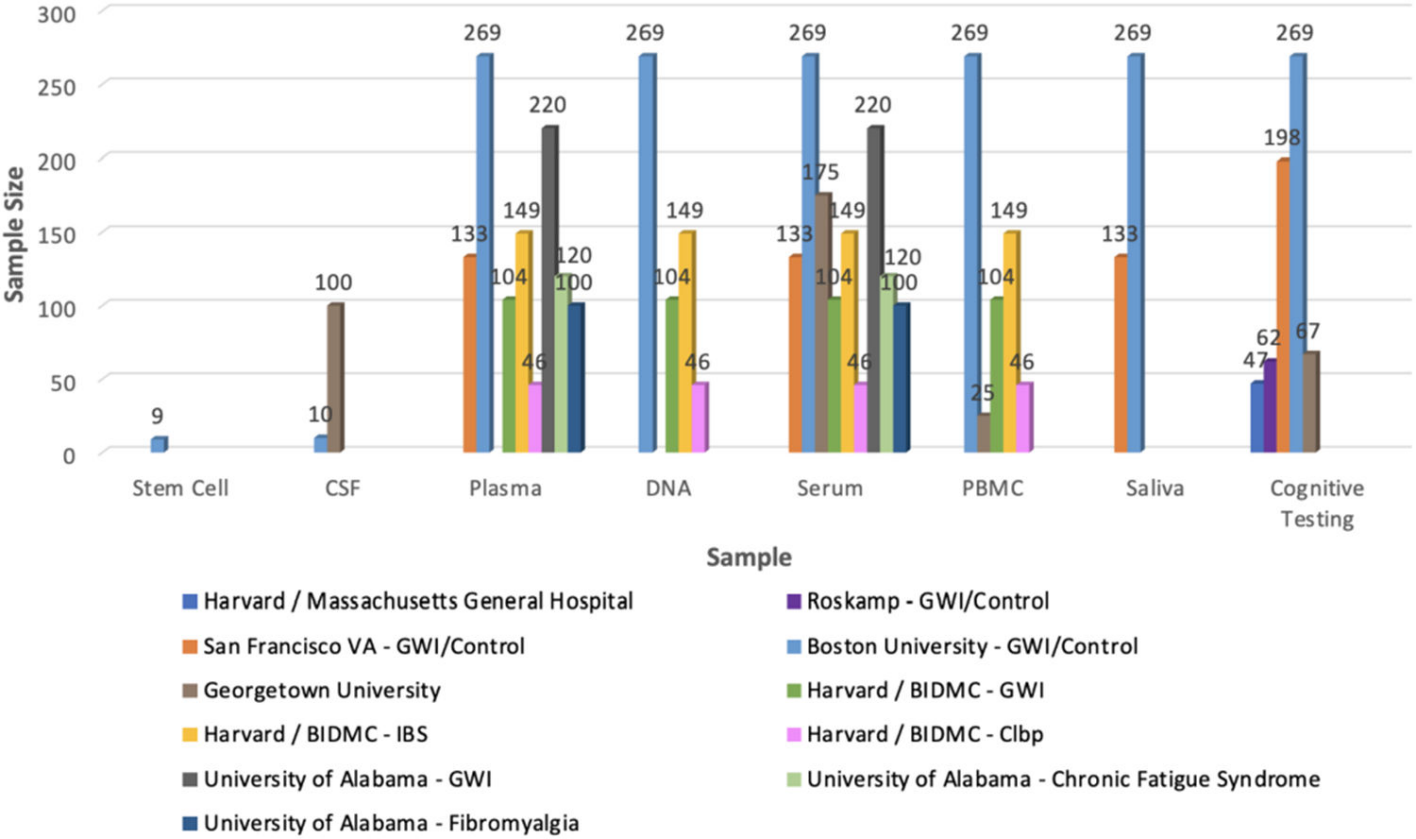
Retrospective BBRAIN Samples and Data Repository.

**Fig. 3.**
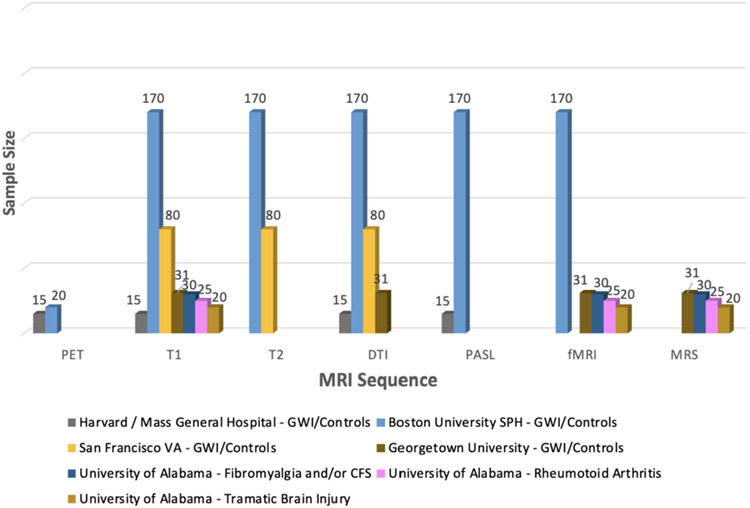
BBRAIN MRI and PET Imaging Repository.

One of the BBRAIN's primary objectives is to establish a retrospective biorepository network by data mining from existing BBRAIN collaborators’ stored specimens, cognitive data, and brain imaging data from study participants who have consented to share these data and samples for future studies in a de-identified manner. Retrospective resource sites have already provided some stored specimens from prior studies with GW veterans cataloged and made available for within Network and outside of Network investigators. Common data element datasets from these prior studies are also being created to improve power for new analyses. Currently, available samples include blood serum (*n* = 1100), plasma (*n* = 1100), peripheral blood mononuclear cells (PBMCs *n* = 600), DNA (n = 600), human-induced pluripotent stem cells (*n* = 9), cerebrospinal fluid (*n* = 150), cognitive data (*n* = 400), brain imaging data (see Fig. 3 range *n* = 50-280) and corresponding demographic/survey data from retrospective resource sites, including University of Alabama at Birmingham, San Francisco VA, Harvard / Beth Israel Deaconess Medical Center (BIDMC), Georgetown University, Boston University and Drexel University (Fig. 2) [38]. Brain imaging data includes MRI volumetric and diffusion tensor imaging, MR spectroscopy, functional MRI and positron emission tomography (PET) imaging with peripheral benzodiazepine receptor [^11^C]-PBR28 and fluorodeoxyglucose 18F-FDG tracers [7,10,11,44,45]. Although some of these samples have been stored and processed differently, these details will be made available to the requesting research investigators to meet their study needs during a study consultation. In addition, preclinical animal retrospective data and tissue samples are also available for sharing upon request from the CDC/NIOSH and Roskamp Institute GWI animal models. As previously mentioned, data collection for the biorepository is coordinated and quality checked by the network coordinating center.

#### 2.2.4. Prospective resource sites

Subject recruitment for the prospective study is conducted at four prospective sites comprising of Boston University School of Public Health (BUSPH), Miami VA Medical Center / Nova Southeastern University (NSU), Bronx VA Medical Center and San Francisco VA Medical Center (Fig. 1). These sites were chosen due to their access to established GW veteran cohorts and important prior research contributions. Data from the prospective resource sites is being added to the BBRAIN biorepository as subject recruitment accrues.

## 3. Prospective study methods

### 3.1. Participants

This case-control study is recruiting 500 GW veterans encompassing 300 GWI cases and 200 GW veteran controls. GWI cases are determined by the Kansas GWI criteria and the four recruitment sites are over-sampling women veterans from their prior cohorts [4]. Although not exclusion criteria, smoking history, medication use, and other demographic and health outcomes are being collected and available to requesting investigators.

### 3.2. Inclusion/exclusion criteria

Study eligibility includes deployment to the Persian Gulf in the 1990-1991 Gulf War without any medical exclusions required for participation. To meet case criteria, the individual needs to endorse symptoms in three of six health symptom domains: pain, fatigue, neurological/cognitive/mood, skin, gastrointestinal, and respiratory [4]. If an individual does not meet the Kansas criteria and has no exclusionary conditions, they are categorized as a control. The criteria for prospective study participants is determined by using the Kansas GWI case definition [4]. Veterans are excluded from being considered GWI cases or controls for the Kansas criteria if they report being diagnosed by a physician with medical or psychiatric conditions that would otherwise account for their symptoms or interfere with their ability to report their symptoms. The Kansas exclusion criteria encompass conditions such as diabetes, heart disease other than hypertension, stroke, lupus, multiple sclerosis, cancer, liver disease, chronic infection, or serious brain injury. Veterans are also excluded if they report being diagnosed with schizophrenia or bipolar disorder or if they have been hospitalized in the past 5 years for alcohol/drug dependence, depression, or post-traumatic stress disorder (PTSD). Potential participants are screened by telephone to determine whether they meet inclusionary or exclusionary criteria for study participation [4]. Additionally, during the phone screen eligible participants are categorized as a case or control based on the Kansas GWI case criteria [4]. Although Kansas criteria are primarily used for comparing study outcomes, the CDC chronic multi-symptom illness case criteria are also obtained for all study participants [5]. These criteria include symptoms in two out of three symptom domains including fatigue, mood-cognition and pain [5].

### 3.3. Methods

The study protocol for the prospective resource site clinical case-control study consists of five components:

1. Neuropsychological testing: Measures from a previously validated assessment of cognitive function and common data elements in GW veterans are included to assess cognitive outcomes [15,35,46-48]. The neuropsychological test battery assesses the functional domains of attention and executive abilities, psychomotor function, visuospatial skills, memory, general intellectual abilities and mood. The battery includes tests shown to have high specificity and sensitivity for detecting changes in neuropsychological functions between veterans with and without GWI and which were recommended to be used across studies as common data elements [35,47].
2. Surveys: The set of surveys administered were all included in the GWI common data elements and collect data on health symptoms, neurotoxicant exposures, mood and quality of life. Clinical assessments include the Pittsburgh Sleep Quality Index (PSQI), Visual Analog Scale (VAS) for pain, Kansas Gulf War and Health Questionnaire, Multidimensional Fatigue Inventory (MFI-20) questionnaire, MOS Short Form 36-veteran version (SF-36V), and Profile of Mood States (POMS), as well as the medical conditions checklist. Additional surveys that were also included in the exposure assessment common data elements include the Kansas Gulf War Experiences and Exposures Questionnaire and the Structured Neurotoxicant Assessment Checklist (SNAC) [49-54].
3. Blood draw: Approximately 79 mL of blood are drawn from the participant for local lab clinical testing, immune biomarker lab testing, and biorepository storage. Fasting blood samples are collected by venipuncture by a trained phlebotomist in the morning. A small amount of blood is analyzed at the local labs for complete blood count, lipid panels, thyroid stimulating hormone, antinuclear antibodies, and rheumatoid factor. The remaining blood samples for each participant are shipped to Nova Southeastern University for sample processing and storage in the biorepository. Blood samples are analyzed to measure plasma cytokine levels, complete blood count, and RNA extraction is performed from PBMCs collected. PBMCs isolated from sodium heparin and EDTA tubes are stored in liquid nitrogen for cryopreservation. RNA isolated from PBMCs is aliquoted and stored at −80 °C. All data from these analyses will be made available as part of the biorepository. Whole blood and blood derivatives such as serum, heparin and EDTA plasma are prepared at various volume aliquots and frozen at −80 °C for storage.
4. Saliva sample: Approximately 6 mL of saliva are collected throughout the study visit. These samples are collected at four different time points: after the participant consent form is signed, after the blood draw and physical exam, after completion of surveys, and at the end of the visit. Three of the collections are performed when the participant is in a fasting state. These samples are used to measure salivary cortisol levels and are stored in a −80 °C freezer. An OGR-600 tube for saliva sample collection is also performed upon fasting for DNA analysis and stored at room temperature before shipping to EM Papper Lab at NOVA Southeastern University. All saliva samples are batch shipped to Nova Southeastern University for planned assessment, cortisol analyses and DNA extraction. The remaining saliva samples are also aliquoted down and frozen for biorepository sample requests. Cortisol and DNA will be made available in the biorepository.

#### 3.3.1. Sample processing methods

**Fig. 4.**
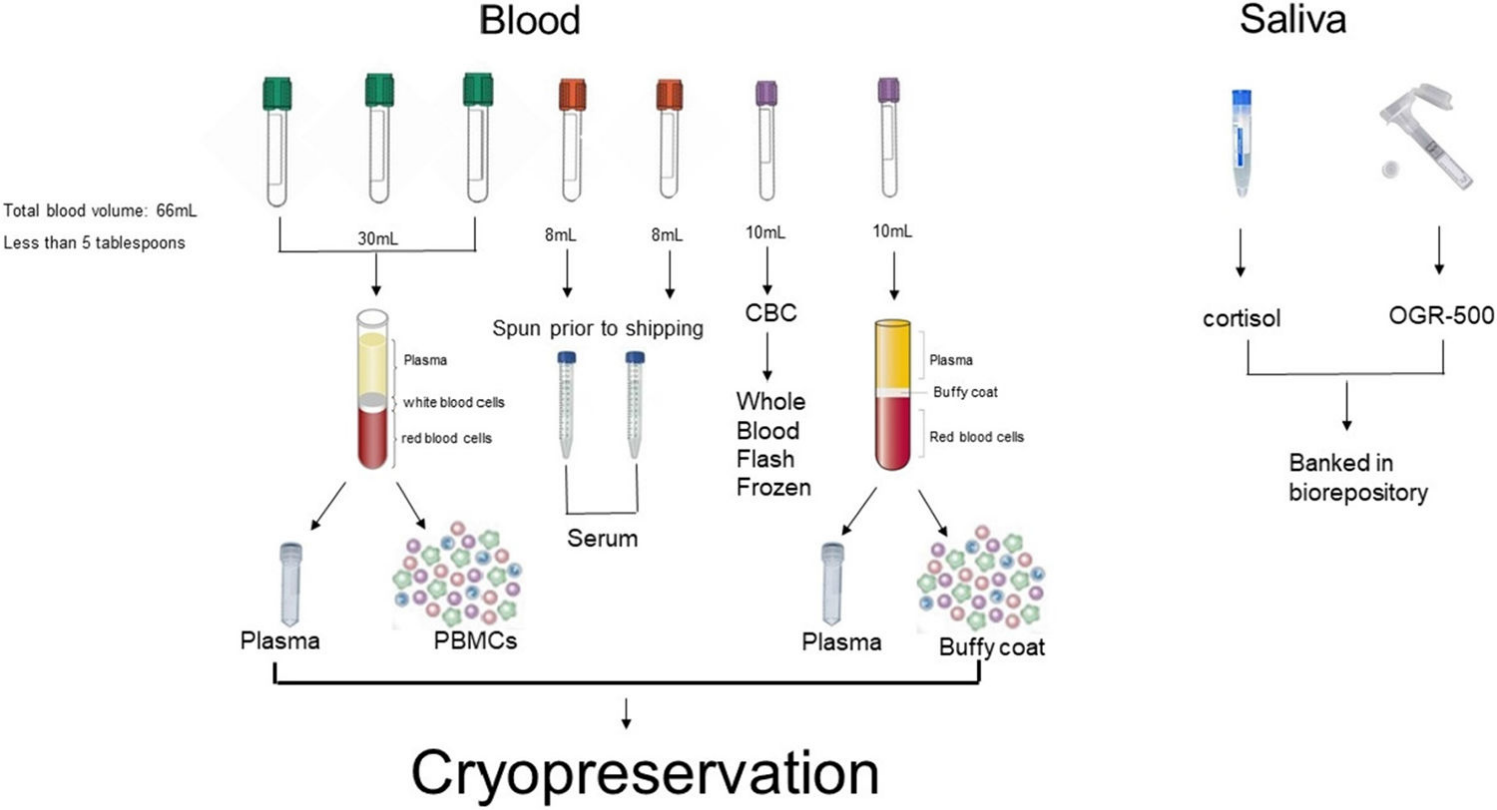
Blood and saliva aliquot scheme.

Blood samples are shipped on the day of collection at room temperature overnight to the EM Papper laboratory at Nova Southeastern University. As previously mentioned, saliva samples are batch shipped on dry ice, from storage at each prospective site's local storage location. Samples received by the biorepository laboratory at Nova Southeastern University are processed within 2 h of delivery. This includes an immediate quality check of the condition of sample tubes, the outer and inner packaging, and the associated chain of custody that accompanies the sample. Acceptable samples are then accessioned by the biorepository team and entered into the Laboratory Data Management System. This allows for the tracking of barcoded aliquots from each phase of the process, including storage conditions and requirements, and will allow expeditious processing of sample requests. Primary tubes are processed to isolate the blood derivatives: plasma, serum, PBMCs, buffy coat, and red blood cell pellets. Aliquots will be created per the aliquot scheme (see Fig. 4) and tracked for their temperature and location. Each of these blood derivative type are stored at its optimal temperature per standard protocol for the EM Papper laboratory at Nova Southeastern University. Specific blood processing methods are listed below.

##### 3.3.1.1. Separation of plasma from the cellular fraction.

Whole blood samples are processed as described below to obtain a buffy coat fraction and plasma for cryopreservation. In the area designated for processing blood, the whole blood (collected in tubes containing an anticoagulant such as ethylene-diamineteraaceticacid-EDTA or Heparin) is fractionated by centrifuging at 2000 x g for 10 min at room temperature. This separates the blood into three visible layers (Fig. 4). The upper layer, the plasma layer, is generally clear or pale yellow in color. The second layer is a narrow grayish white interface band representing the “buffy coat” or leukocyte fraction. The third or bottom layer is dark red and consists of the erythrocytes or red blood cells. Using an appropriate disposable transfer pipette, the plasma layer is aspirated off down to approximately 1 mm from the buffy coat layer taking care not to disturb the leukocyte or buffy coat layer. All plasma is expelled from the pipette into a plasma collection tubes. Recovered plasma is aliquoted and placed into labeled cryovials. The barcoded cryovials are placed in appropriate storage units for long-term storage in −80 °C freezers at the EM Papper laboratory at Nova Southeastern University.

##### 3.3.1.2. Recovery of white blood cells.

After removing the plasma layer, removal of buffy coat is performed. To isolate PBMCs, sufficient quantity of PBS is added to bring blood back to its original whole blood volume and mixed gently to continue PBMC processing. A transfer pipette is used to transfer all of the blood into a 15 mL tube containing 3 mL Ficoll-Hypaque solution. The tube is centrifuged (without a brake) at 2250 x g for 25 min at 25 °C. Using a sterile serological or transfer pipet, all cells are collected at the cloudy white interface taking care not to aspirate any more separation medium solution than necessary. The collected cells are transferred from one conical centrifuge tube to a single corresponding, pre-labeled, sterile conical centrifuge tube. After centrifugation of the wash step, cells are resuspended in 10 mL of PBS for cell count and viability using the Beckman Coulter ViCell Counter. For this study, 5 × 10^6^ cells/ml per vial are aliquoted in final freezing solution of 70% RPMI 1640 with 20% fetal bovine serum and 10% dimethyl sulfoxide (DMSO) as a cryoprotectant added. The cryovials are placed in the appropriate storage units at −80 °C for short-term storage. For long-term storage, cells stored in freezing vials are transferred into the liquid nitrogen cryopreservation tanks and their location is mapped and recorded.

##### 3.3.1.3. Separation of serum from blood samples.

Blood is drawn into BD Vacutainer^®^ SST^™^ Venous blood collection tube with separator gel. This tube is spun down prior to shipping to the EM Papper laboratory. Serum sample above the gel separator is collected and stored in barcoded cryovials at various aliquot volumes at −80 °C for long term storage.

Quality Assurance and Assessment protocols take place before, during and after the samples are isolated from primary tubes and placed into the biorepository. Samples for all four prospective resource sites are processed with the same protocols.

#### 3.3.2. Common issues of aliquot size

Biorepositories face a lack of predictability in future research direction, limited resources in space and maintenance manpower, as well as new technology innovations, which makes planning a biorepository difficult. It is imperative to create a versatile sample aliquot scheme in order to combat these challenges. However, repositories commonly will need to be flexible with remaining sample subsets should circumstances change, while avoiding “freeze thaw cycles” that are potentially damaging to certain proteins in serum or plasma. While large aliquots of 2 mL or greater can reduce maintenance costs and space requirements, small aliquots are the versatile option that allow for greater flexibility when fulfilling sample requests. This laboratory takes a different approach, setting up large numbers of small aliquots (0.5 and 0.25 mL) while still creating a small number of large aliquots (1.0 mL) for longer term storage (Fig. 4; Table 1).

5) Home specimen and data collection

Home collection by study participants includes urine, stool samples, and Fitbit data collection. Specific methods and details are listed below.

##### 3.3.2.1. Fitbit data.

Each participant is asked to wear a study-provided Fitbit activity monitor for 7 consecutive days. On the eighth day, participants are expected to extract their sleep quality and heart rate variability data and upload the results to the network coordinating center through a secure link. An instructional manual is provided to the participant for proper data extraction. All Fitbit data are made available for sharing in the repository.

##### 3.3.2.2. Urine samples.

Urine sample kits are provided to the participant with comprehensible instructions during the in-person study visit to complete at home and mail back in prepared pre-paid packaging. Participants are expected to collect at least 20 mL urine during their first morning void. Urine samples from all study sites are overnight shipped with an ice pack to maintain cold chain transport to the Boston University Medical Campus for aliquoting and storage. Once received, the urine samples are aliquoted, frozen and stored in the repository for later urinalysis, sharing and sample requests (Table 1).

##### 3.3.2.3. Stool samples.

Stool sample kits are provided to the participant during the in-person visit with clear instructions to complete the at home stool sample kit and mail it back in a prepared pre-paid packaging. Participants are expected to collect two tubes of stool. Stool samples from all study sites are overnight shipped to Boston University Medical Center for storage. Samples are stable for 15 days at room temperature and very well preserved over a long period of time at −4C. Samples will be stored at the Boston University Medical Center Laboratory and available for collaborative research and for requests through the repository.

## 4. Data sharing

The BBRAIN biorepository has a responsibility to provide access to samples for pilot work and other initiatives to further the field. This is accomplished by a three-stage approval process for any data or samples that exist as part of this project and/or the biorepository. This process is streamlined through a web-based data request platform created by the network coordinating center. The first steps involve a potential investigator contacting the project PI to determine validity of an idea. Once initial conversations are considered positive, the potential investigator submits a proposal, along with a sample or data request form, to the Steering Committee. This committee then assesses the proposal for feasibility (samples must be available, technology/assays must have a reasonable chance of success), duplicative effort (avoids overlap from funded portion of the grant or previously approved requests), and sample volume requested (protects the integrity of the aliquot scheme, i.e. requesting 1 mL when 200uL would suffice). Finally, the request is reviewed by the Steering Committee for further consideration and final approval or denial. There is no cost for the sharing of samples from the repository except the cost of shipping the samples to the approved requestors.

### 4.1. How to request samples and data

As previously described, the BBRAIN Repository Network provides samples and data from newly collected whole blood, RNA, DNA, plasma, serum, saliva, stool, and urine samples for 500 GW veterans (300 GWI cases, 200 controls) in addition to demographic surveys and cognitive test data. In addition, a repository of previously collected demographic and health survey, clinical (cognitive testing, MRI data) and preclinical data (animal tissue) has been compiled from the 15 participating GWI investigators and made available to the BBRAIN repository for data mining and sharing. 9 lines of hiPSCs collected from 5 GW-veterans with GWI and 4 from those who did not develop GWI are available upon request. As previously outlined, the Network Coordinating Center organizes approvals with the steering committee members in consultation with the biorepository contributor sites. The site for requesting prospective and retrospectives biospecimens, brain imaging and other health symptom or cognitive data is available at https://wwwapp.bumc.bu.edu/BEDAC_BBrainRetro.

## 5. Discussion

The BBRAIN is the first GWI repository network designed to gather and store samples, provide new data and to mine data from prior studies of difficult to obtain samples (CSF, PET, MRI imaging). In doing so, BBRAIN has grown, and continues to shape and fill the need for an easily accessible biospecimen repository in the field of GWI research. An additional primary objective of this biorepository network has been to determine minimal data elements using a common data platform from retrospective studies, thus creating centralized resource websites for BBRAIN researchers and other interested GWI researchers seeking to obtain repository samples and data for analyses. The task has been completed, with common data elements for symptom and system domains now identified. To date, BBRAIN has published recommended common data elements for neuropsychological and other outcomes [15,47]. Importantly, the BBRAIN prospective sample and data collection is consistent with the same elements of the common data recommendations and will therefore provide additional validation of nearly all of the common data elements.

BBRAIN will build upon the initial progress of the Boston GWIC biorepository. It has been stated that the success of a biorepository is not in how many samples are collected but in how many samples are shared that lead to important new results [55-58]. To date, BBRAIN has shared samples with five research investigators including cerebrospinal fluid, serum and plasma samples, PET brain imaging, diffusion MRI brain imaging data and cognitive outcomes [59]. Building on this strong foundation, BBRAIN aims to provide the infrastructure, scientific expertise, biological specimens and collaborative nature to vastly speed up objective biomarker discovery and treatments for ailing veterans with GWI. The robust infrastructure of the BBRAIN repository network will serve as a key resource for the GWI research field.

## 6. Conclusion

BBRAIN, built from a strong foundation of collaboration and need for a biorepository in the community of GWI research, aspires to provide the scientific resources to identify more definitive biomarkers for GWI. The team approach of sharing samples will lead to faster identification of diagnostic tests for GWI and targeted personalized medicine treatments for ill veterans. For veterans who have remained ill for over 30 years, the importance of quickly identifying diagnostic tests and effective treatments for GWI cannot be overstated.

## Figures and Tables

**Table 1:** BBRAIN blood and saliva aliquot and storage procedures.

Sample type	Tube type/color	# tubes	Tube volume (ml)	Total volume (ml)	Product	Expected product volume - biobank	Aliquot scheme	Test
	(3) Green NaHeparin	3	10	30	Blood, PBMC, Plasma	60 × 10^6 PBMC; 12mL Plasma	PL2: 4 × 1 mL, 4 × .5 mL, 24 × .25mL; CEL: 12 × 5 × 10^6/mL	NKCC, NPY, Nanostring
Blooddraw	(2) Red Tiger Middle SST Tube	1	8	16	Serum	3 mL	2 × 0.5 mL; 8 × 0.25 mL	Hormone specific TBD
	(2) Purple EDTA	2	10	20	Blood, Serum	6 mL	4 × .5 mL; 16 × .25 mL	CBC, Flow, Cytokines
	(1) OGR-500	1	2	2	Saliva	2 mL	N/A	SNP, TBD
Saliva	(4) Salimetrics’ Cryovials	4	2	8	Saliva	8 mL	2 × 1 mL; 12 × .5 mL	Salivary Cortisol, TBD

## Abbreviations:

BBRAIN: Boston Biorepository, Recruitment and Integrative Network for Gulf War Illness
GW: Gulf War
GWI: Gulf War Illness
GWIC: Gulf War Illness Consortium
VA: Veterans Affairs
